# Phase I clinical, pharmacokinetic and pharmacodynamic study of SB939, an oral histone deacetylase (HDAC) inhibitor, in patients with advanced solid tumours

**DOI:** 10.1038/bjc.2011.13

**Published:** 2011-02-01

**Authors:** A R A Razak, S J Hotte, L L Siu, E X Chen, H W Hirte, J Powers, W Walsh, L-A Stayner, A Laughlin, V Novotny-Diermayr, J Zhu, E A Eisenhauer

**Affiliations:** 1 Drug Development Program, Department of Medical Oncology and Haematology, Princess Margaret Hospital, Suite 5-718 (5th Floor), 610 University Avenue, Toronto, Ontario, M5G 2M9 Canada; 2 Department of Medical Oncology and Haematology, Juravinski Cancer Centre, Concession Street, Hamilton, Ontario, L8V 5C2, Canada; 3 NCIC Clinical Trials Group (NCIC-CTG), Cancer Clinical Trials Division, Cancer Research Institute, Queens University, 10 Stuart Street, Kingston, Ontario K7L 3N6, Canada; 4 S^*^BIO Pte Ltd, 1 Science Park Road, #05-09 The Capricorn, Singapore Science Park II, 117 528, Singapore

**Keywords:** histone deacetylase, phase I, toxicity, pharmacokinetic, pharmacodynamic

## Abstract

**Background:**

SB939 is an orally available, competitive histone deacetylase (HDAC) inhibitor selective for class I, II and IV histone deacetylases. Preclinical evaluation of SB939 revealed a profile suggesting improved efficacy compared to other HDAC inhibitors. This phase I study was carried out to determine the safety, dose-limiting toxicity, recommended phase II dose (RPTD), as well as pharmacokinetic (PK) and pharmacodynamic (PD) profiles of SB939 in a daily × 5 schedule in advanced solid tumours.

**Methods:**

Sequential dose-escalating cohorts of patients were enrolled into 8 dose levels. At dose level 1, SB939 was taken on days 1–3 and 15–17 every 4 weeks, then on days 1–5 and 15–19 for other dose levels. Detailed PK sampling was performed in cycle 1, days 1 and 5. Peripheral blood mononuclear cells (PBMCs) were collected on cycle 1 at various time points for determination of acetylated histone H3 (AcH3) levels.

**Results:**

In total, 38 patients received a total of 96 cycles of treatment. The maximal administered dose was 90 mg and the RPTD was 60 mg given 5 consecutive days every 2 weeks. The most frequent non-hematologic adverse events (AEs) of at least possible attribution to SB939 were fatigue, nausea, vomiting, anorexia and diarrhoea. Pharmacokinetic analysis showed dose-proportional increases in AUC across the doses evaluated. Elimination half-life was 5.6–8.9 h. There was no clear relationship between AcH3 changes and dose level or anti-tumour response.

**Conclusions:**

SB939 is well tolerated in patients with advanced solid tumours. The RPTD of this drug is 60 mg on a schedule of 5 consecutive days every 2 weeks. The toxicities of SB939 are consistent with other HDAC inhibitors.

Epigenetic changes that occur through chromatin modulation regulate the accessibility of gene promoters to the transcription and replication machinery (Strahl and Allis, 2000; Johnstone and Licht, 2003). Chromatin modulation and its associated effects on gene expression are controlled by opposing effects of two families of enzymes: histone acetylase transferases (HATs) and histone deacetylases, known as HDACs (Chan *et al*, 2001; Carey and La Thangue, 2006). Histone deacetylation is an important epigenetic event implicated in the development and progression of cancer and provides an attractive anti-tumour therapeutic platform (Pandolfi, 2001; Timmermann *et al*, 2001; Chambers *et al*, 2003). The HDAC family is divided into zinc-dependent (class I, II and IV) and zinc-independent, nicotinamide-adenine dinucleotide-dependent (class III) categories (Stimson *et al*, 2009). At present, most HDAC inhibitors being developed as anti-cancer agents target class I, II and IV enzymes and there is increasing interest in the class III family. The accumulation of acetylated proteins through HDAC inhibition results in a variety of cell type-dependent responses, such as differentiation, induction of cell cycle arrest, apoptosis, as well as altered patterns of gene expression.

SB939 (3-[2-butyl-1-(2-diethylaminoethyl)-1*H*-benzoimidazol-5-yl]-*N*-hydroxyacrylami-hydrochloride) is an orally available, competitive inhibitor of HDAC. *In vitro* studies showed that SB939 has >1000-fold selectivity for class I, II and IV HDACs compared with class III HDACs with no effects on other zinc-binding enzymes (Novotny-Diermayr *et al*, 2010). The *in vitro* evaluation of SB939 also showed significant anti-proliferative activities against a wide variety of cell lines. Immunoblotting techniques showed that SB939 treatment of cancer cells results in the accumulation of acetylated histone H3 (AcH3) and acetylated *α*-tubulin, as well as increased expression levels of the cyclin dependant kinase inhibitor p21 (Novotny-Diermayr *et al*, 2010).

SB939 has favourable pharmacokinetic (PK) properties in animal pre-clinical models, with >4-fold increased bioavailability and 3.3-fold longer half-life compared with suberoylanilide hydroxamic acid (vorinostat), another HDAC inhibitor active in human malignancies. SB939 also demonstrated prolonged drug accumulation and more sustained inhibition of histone deacetylation in tumour tissues compared with vorinostat in preclinical experiments. In HCT-116 cells, SB939 was more effective than vorinostat. These excellent PK and pharmacodynamic (PD) properties translated into a dose-dependent anti-tumour efficacy in several experimental solid tumours models, including xenograft models of colon (HCT-116), ovarian (A2780) and prostate (PC3, DU145) carcinomas, as well as murine models of acute myeloid leukaemia (MV4-11, HL-60) and B cell lymphoma (Novotny-Diermayr *et al*, 2010).

Based on the relevance of HDACs as cancer therapeutic targets, and the encouraging preclinical profile of SB939, the NCIC CTG conducted a phase I trial in patients with advanced solid tumours. The primary objective was to determine the RPTD, safety and tolerability of SB939 given on a daily × 5 every other week schedule. Secondary objectives included the assessments of PK and PD changes in peripheral blood mononuclear cells (PBMCs) and preliminary anti-tumour efficacy. The choice for the human starting dose of 10 mg was based on 1/10th of the no observed adverse effect level of the dog species at 60 mg m^−2^ per day (3 days per week in a 28-day cycle), rounded to the nearest available fixed dose capsule size. In this study, the every other week schedule was evaluated because trials of other HDAC inhibitors suggest fatigue is a common AE that can prohibit continuous dosing. Therefore, a schedule of continuous 5-day dosing on alternate weeks was deemed appropriate to try to avoid dosing interruptions. In addition to this NCIC CTG study, another phase I trial evaluating three times weekly dosing 3 out of 4 weeks was undertaken by investigators in Singapore (Yong *et al*, 2008, 2009).

## Methods

### Patient eligibility

Patients were eligible if they had histologically or cytologically documented advanced solid malignancy, refractory to standard therapy or for which conventional therapy was not effective. Other key eligibility criteria included: age ⩾18 years; Eastern Cooperative Oncology Group performance status 0–2; adequate haematologic, hepatic and renal functions (absolute neutrophil count ⩾1.5 × 10^9^ l^−1^, platelets ⩾100 × 10^9^ l^−1^, bilirubin ⩽upper limit of normal, aspartate transaminase (AST)/alanine transaminase (ALT) ⩽2.5 times upper limit of normal or <5 times upper limit of normal if documented liver metastases, creatinine ⩽1.2 times upper limit of normal (or calculated creatinine clearance of ⩾60 ml s^−1^ via Cockroft and Gault formula); adequate cardiac function (LVEF of ⩾50%, Troponin I or T ⩽upper limits of normal and a corrected QT (QTc) interval of ⩽450 ms). Prior therapy, except for HDAC inhibitors, was allowed, but there must be a 4-week interval between previous systemic/radiotherapy and initiation of study drug. Patients with documented central nervous system metastasis, pathologic arrhythmia requiring active treatment within the past 12 months or with significant co-morbidities were excluded from the study. As well, patients on agents with a known risk of Torsades de Pointes were excluded. The institutional review board of both participating centres approved the study, which was conducted in accordance with the federal and institutional guidelines.

### Study design and patient evaluation

This was a two-centre, single-agent, open-label dose-escalation phase I study in which SB939 was administered orally in an empty stomach (⩾1 h before a meal or 2 h after). SB939 was administered in an empty stomach as there were no data available on the effect of food intake on drug absorption at the time the trial was initiated. Study drug was administered at a starting dose of 10 mg on days 1–3 and 15–17 for the first dose level while for subsequent dose levels, treatment was administered on days 1–5 and 15–19, within a 28-day cycle. No intra-patient dose escalation was allowed. Dose escalation followed the standard 3+3 rule, but the protocol did allow up to four patients to be entered in the first stage of accrual at each dose level. The maximum administered dose was defined as the dose level at which ⩾2/3 or ⩾2/6 patients experienced dose-limiting toxicity (DLT). The next lower dose would normally be declared the RPTD for expansion to confirm the tolerability and PK and/or PD relationships at this dose level.

In cycle 1, patients were seen on days 1, 5, 8, 15, 19, 22 and weekly in subsequent cycles for laboratory studies and assessment of toxicity. As HDAC inhibitors have been associated with cardiac effects, electrocardiographs (ECGs) were undertaken pre- and post-dose in cycle 1 on days 1, 5, 15, 19 and on day 1 of subsequent cycles. In addition, troponin levels were obtained on days 1, 5, 15 in cycle 1 and on day 1 of subsequent cycles. Toxicity was graded using the National Cancer Institute Common Terminology Criteria for Adverse Events version 3.0 (http://ctep.cancer.gov/protocolDevelopment/electronic_applications/ctc.htm#ctc_30). Dose-limiting toxicities were defined as AEs occurring during the first cycle of SB939 administration and fulfilling one of the following criteria: grade 4 neutropenia lasting ⩾5 days or associated with fever or infection; thrombocytopenia ⩾grade 3 or thrombocytopenic bleeding ⩾grade 3; nausea/vomiting ⩾grade 3 despite anti-emetic therapy; other major organ toxicity of ⩾grade 3 and delay in starting cycle 2 of >2 weeks or missing two or more consecutive doses within a cycle due to treatment-related AEs of laboratory abnormalities.

For patients with measurable disease at baseline, response was assessed using the Response and Evaluation Criteria in Solid Tumours version 1.0 (Therasse *et al*, 2000).

### Dose modification

Patients who experienced any DLT were delayed by 1-week intervals until recovery and could continue on study drug with a reduction of one dose level. If no recovery occurred after a delay of 2 weeks from the next scheduled 5-day treatment, patients were discontinued from protocol treatment. In addition, patients must have an absolute neutrophil count of ⩾1.5 × 10^9^ l^−1^ and a platelet level of ⩾100 × 10^9^ l^−1^ before day 1 and 15 dosing. SB939 was held if grade 2 QTc interval prolongation occurred, and resumed upon recovery to baseline/grade 1, while a grade 3/4 prolongation of QTc interval would mandate drug discontinuation.

### Duration of study treatment

Patients could receive repeated cycles of treatment until disease progression, unacceptable AE(s), patient's decision to withdraw from the study, or changes in the patient's condition including intercurrent illness rendering the continuation of study treatment unacceptable.

### Pharmacokinetic analysis

Blood samples for evaluation of SB939 PK were collected in cycle 1, days 1 and 5 (days 1 and 3 for dose level 1) before dosing, then 0.5, 1, 1.5, 2, 3, 4, 6, 8 and 24–30 h after dosing. Pre-dose PK assessment was also performed on day 15 of treatment.

Whole blood was collected into a 3-ml blood tubes containing K2EDTA anti-coagulant and centrifuged at 1000 g for 10 min at 4°C. Plasma was kept as an aliquot and frozen at −80°C. Levels of SB939 in plasma were determined using a validated LC–MS/MS method (Novotny-Diermayr *et al*, 2010). The samples were centrifuged at 1200 g for 10 min at 4°C, and the supernatant was stored at −80°C until analysis at MPI Research, Mattawan, MI, USA. Pharmacokinetic parameters were calculated by a non-compartmental method using WinNonlin 4.0 software (Pharsight Corporation, Mountain View, CA, USA). Peak plasma concentration (*C*_max_) and time to peak concentration (*T*_max_) were estimated from the plasma drug concentration–time curve. The elimination half-life (*T*_1/2_) during the log-linear terminal phase was calculated from the elimination rate constant determined by a linear regression analysis. The area under the concentration time curve extrapolated to infinity (AUC_0−∞_) was calculated by the log-linear trapezoidal method for the observed values, with extrapolation to infinity including at least three points on the terminal phase.

### Pharmacodynamic analysis

Based on the preclinical data showing parallel changes in AcH3 in PBMCs and tumour tissue, PBMCs were selected for study in the phase I trial (Novotny-Diermayr *et al*, 2008; Yong *et al*, 2009).

Whole blood samples for PD evaluation were taken in cycle 1, days 1 and 15 at pre-dose followed by 3 and 24 h post-dose. For each sample, blood was collected at room temperature in an 8-ml BD Vacutainer CPT tube (Becton, Dickson and Company, Franklin Lakes, NJ, USA). Blood samples were processed in a refrigerated centrifuge within 30 min of collection to preserve histone acetylation status. PBMCs were extracted from blood, processed to pellet form and stored at −80°C. Levels of AcH3 in PBMCs were determined using a validated western blot technique (Novotny-Diermayr *et al*, 2008).

## Results

### Patient demographics, dose escalation and recommended phase II dose

Thirty-nine patients were registered to this study but one patient cancelled registration before treatment. The 38 treated patients (Table 1) completed a total of 96 cycles of SB939 (median, 2; range, 1–12). Eight dose levels ranging from 10 to 90 mg were evaluated (Table 2, dose levels detailed in a chronological order). At the time of this report, all patients are off study treatment.

At the 10 mg dose level (5 days every 2 weeks), a patient was observed to have a dose-limiting grade 3 bilirubin elevation, and this event was initially attributed to drug. Thus, this dose level was expanded by a further three patients, with no further DLTs observed. Although the rise of bilirubin was temporally related to SB939 administration, it was later deemed unrelated to treatment and found to be related to a blocked stent.

At the 20 mg dose level, one patient experienced grade 3 myositis, so again the dose level was expanded with no further DLTs observed. No DLTs were seen at the 30, 50 and 70 mg dose levels during the escalation phase of the trial. At the 90 mg dose level however, two patients were treated and experienced significant toxicity during cycle 1. The first patient had grade 3 fatigue and vomiting, while the dose for the second patient had to be reduced to 70 mg on days 15–19 due to intolerable grade 2 nausea, vomiting and fatigue. It was felt that further dosing of patients at this level was inappropriate and 90 mg was deemed the MAD. As per protocol, the dose level of 70 mg was then re-opened for expansion. At this dose level, the first patient had grade 4 thrombocytopenia with a delay of day 15 dosing, while the second patient had grade 3 fatigue. The third patient in the expansion cohort had grade 2 nausea and vomiting and was unable to complete the first week of treatment. These events suggested that 70 mg was poorly tolerated so an intermediate dose level of 60 mg was open for evaluation. Seven patients were entered for evaluation. One patient came off study after 1 week due to grade 3 ALT rise. This patient, tested negative for viral hepatitis serology and autoimmune workup, had a prior history of transaminitis with other drugs but a relationship with SB939 could not be excluded. Aside from the patient with elevated LFTs, no other DLTs were seen. This dose level (60 mg) was thus concluded to be the RPTD.

### Safety and compliance

All 38 treated patients were evaluable for non-haematologic, haematologic and biochemical toxicities. The most frequently reported AEs of all grades and those grades 3 or higher, separated by dose levels and of at least possible relationship to SB939, are described in Table 3 for non-haematologic events. The most frequently reported, related non-haematologic AEs were fatigue (53%), nausea (39%), vomiting (29%), anorexia (29%) and diarrhoea (18%). The majority of these were grade 1 or 2 events, however 4 patients experienced grade 3 fatigue, one patient had grade 3 diarrhea while one patient had grade 3 vomiting. Other grade 3 related events included myositis and syncope (one patient each).

In terms of cardiac AEs, four patients had grade 2 QTc prolongation documented in study, three in patients receiving a dose level of 60 mg or higher. One had a single elevated value on day 1 cycle 1 that was not confirmed and received for the remainder of their 5-day treatment at an unchanged dose. Two patients had elevated QTc documented on day 5 of therapy, so following the scheduled off-therapy week, the second 5-day treatment was given at a reduced dose with no recurrence of QTc prolongation. The fourth patient was complex. On Day 1 cycle 7 post-dose ECG showed frequent premature ventricular contractions and some episodes of bigeminy. This lasted about 48 h and on investigation it appeared that patient had had similar episodes over the past 4 years. No cause was found for it. During periods of arrhythmia, LVEF fell (values 20–39% compared with baseline of 55%). Troponin remained normal but QTc was slightly elevated in some of the ECG tracings (in nine tracings done in the 10 h after day 1 dose, three patient had grade 1 elevation and one patient had grade 2 (maximum QTc 475 ms)). Patient was asymptomatic throughout and was removed from the study for progressive disease at the same time. It was unclear if this episode was study drug related.

One patient experienced paroxysmal atrial tachycardia (grade 3, unlikely related to study drug) 27 days after his last dose of SB939 associated with chest pain and dizziness. The patient had a slight elevation in troponin, normal QTc and converted to sinus rhythm with carotid massage. The relationship to SB939 was uncertain as the patient had a significant history of coronary artery disease and angina.

Thirty-six of 38 patients had documented anaemia on study (grade 1=25 patients, grade 2=10 patients and grade 3=1 patient). The number of patients experiencing other haematologic AEs increased with dose, particularly at ⩾60 mg compared with dose levels ⩽50 mg, respectively: thrombocytopenia 87 *vs* 22% granulocytopenia 60 *vs* 26% lymphopenia 80 *vs* 57%. Biochemical changes have been modest, all were grade 1 in patients with normal baseline chemistries except one patient with myositis who experienced a grade 3 CPK elevation, 3 patients at 60 mg who had grade 2 raised AST and hyperbilirubinemia and two patients at that same dose level who had grade 3 ALT rise. Table 3 depicts haematologic and biochemical AEs of all grades and those grades 3 or higher.

Dose reductions or omissions were seen occasionally in patients treated from 10–60 mg but were more frequently observed at 70 and 90 mg dose levels (reductions in 2 of 6 and 2 of 2 patients, respectively). At the RPTD of 60 mg (*n*=7), two patients had dose reductions (transaminase rise and QTc prolongation), while two patients had dose omissions (both due to alteration of liver function tests).

### Anti-tumour activity

Thirty-six of the 38 treated patients had measurable disease at baseline. Of these, five patients were not assessable for response because they received one cycle or less of treatment and were not re-imaged. No objective tumour responses were observed. Ten patients of the 31 evaluable (32%) had a best response of stable disease (SD) while 21 (68%) patients had disease progression. Patients with SD had a median duration of disease control for a median of 5.7 months (range 1.8–10.8 months). Three patients, all with history of metastatic colorectal carcinoma had disease stabilisation of >6 months. These patients were treated at dose levels 2, 3 and 4, with disease stabilisation achieved for a period of 8.1, 10.8 and 7.4 months, respectively, before stopping treatment due to evidence of disease progression.

### Pharmacokinetic analysis

**Figure 1:**
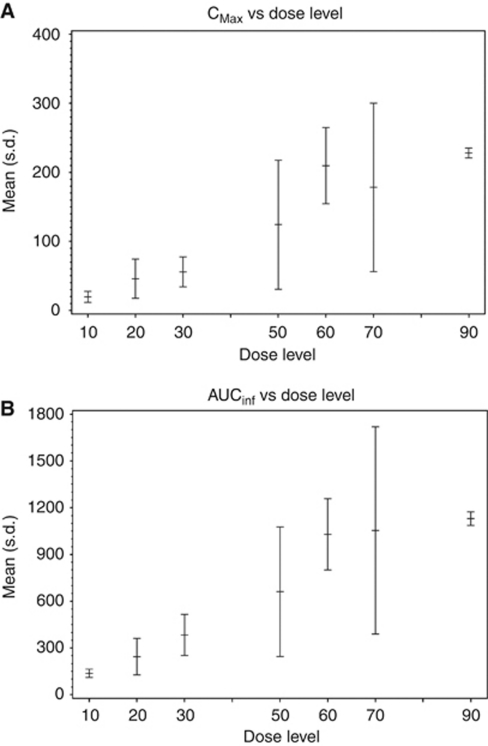
(**A**) Mean *C*_max_ and (**B**) AUC_0–∞_ of SB939 by dose level.

Pharmacokinetic analysis showed SB939 was rapidly absorbed (*T*_max_ range of 1.0–2.5 h) and has bi-exponential disposition. The terminal half-life (*T*_1/2_) ranged between 6 and 9 h. There was no accumulation on Day 5 compared with Day 1. Mean AUC increased dose proportionally between 10 and 90 mg. A summary of the *C*_max_ and AUC_0−∞_ at different dose levels are represented in Figure 1, while the full data on AUC_0−∞_, *T*_1/2_, *C*_max_ and *T*_max_ are detailed in Table 4.

### Pharmacodynamic analysis

Increases in AcH3 were seen in patients treated in study with some patients experiencing maximal increase at 3 h post-treatment and others at 24 h post-treatment. There was no clear dose–PD relationship noted. There were several PBMC samples that were unsuitable for analyses in the mid-dose range, resulting in our inability to define a dose–PD correlation. However, two individuals with the largest changes in levels were treated at the 60 and 70 mg dose levels. No relationship between PD and disease stabilisation or time on study could be discerned.

## Conclusion

This study demonstrates that SB939 administered orally is tolerable in patients with advanced solid tumours. The RPTD was 60 mg daily utilising a 5-day every 2 weeks schedule. The administration of SB939 results in toxicities that included fatigue, nausea, vomiting, anorexia and diarrhea. These symptoms have been observed previously with this class of agents (Marshall *et al*, 2002; Sandor *et al*, 2002; Kelly *et al*, 2005; Ryan *et al*, 2005; Giles *et al*, 2006; Garcia-Manero *et al*, 2008; Siu *et al*, 2008). These toxicities, however, were non-life threatening, and were effectively addressed by dosing delay and dose reduction. While QTc elevations were documented, none were grade 3. More data on the frequency of QTc changes will be known following phase II trials where monitoring during cycle 1 will be undertaken.

Although there was no objective tumour response to treatment, 31% of patients observed with SD (median 5.7 months), most of whom had been heavily pre-treated. There were three colorectal cancer patients with durable period of SD, but the numbers were too small for any meaningful conclusion to be made.

When compared to the study by Yong *et al*, which gave SB939 every other day (three times per week), for 3 out of 4 weeks, our dosing schedule appeared to have somewhat less myelosuppression, but possibly more non-haematological AEs. However, comparison of adverse effects between schedules in a non-randomised setting must be viewed with caution. The schedule selected for phase II evaluation is the every other day schedule, in part because both achieved the same recommended dose and the every other day schedule seemed to have less symptomatic toxicity, but also because more continuous exposure might provide better therapeutic efficacy, based on preclinical schedule data. Furthermore, the every other day schedule explored by Yong *et al* had demonstrated a dose–PD effect, strengthening the rational for choosing their dose schedule over ours in further development of this drug.

The PK evaluations in this study have demonstrated favourable pharmacologic properties of SB939. The drug was rapidly absorbed within after oral administration, with a median *T*_max_ ranging from 1 to 2.5 h. Importantly, the drug had a long elimination half-life in plasma of up to 9 h, which compares favourably with the shorter, 1–2-h half-lives of other HDAC inhibitors such as vorinostat and belinostat (Kelly *et al*, 2005; Steele *et al*, 2008). The PK properties as well as the RPTD of this study were also similar to the trial carried out by our colleagues in Singapore (Yong *et al*, 2008). In that study, the RPTD of SB939 was also 60 mg in patients with solid tumours and the AUC_0−∞_, *T*_1/2_, *C*_max_ and *T*_max_ in that study were comparable to ours.

The PD activity of HDAC inhibition arising from SB939 administration was explored in this study, utilising a validated western blotting method. From cumulative data thus far, histone acetylation does not show a linear correlation with dose. This may be explained in part by the observation that several of the PBMC samples in our study (in the mid-dose range) were not suitable for PD analyses, resulting in the loss of dose–PD information. Despite this, the highest AcH3 levels in our study were seen in patient cohorts treated with higher doses of SB939, demonstrating the proof of mechanism that this agent indeed leads to histone deacetylation in the clinical setting. Furthermore, our results also parallel the findings of our colleagues in Singapore where a relationship between AcH3 levels and dose was observed (Yong *et al*, 2009).

In our study, there is also a lack of correlation between AcH3 levels and anti-tumour activity. Although the loss of PD data set point may contribute to this finding, there is increasing evidence of non-histone protein substrates on which HDAC inhibitors exert their anti-cancer effects. These include p53, Ku70, Hsp90, Stat3, NF-*κ*B and tubulin and the modulation of these substrates have a role in regulating cell proliferation and survival (Lin *et al*, 2006). Furthermore, phase I trial is not the setting in which one expects to observe neither sufficient anti-tumour activities nor clear PD-anti-tumour efficacy correlations.

Despite the promising preclinical activity of HDAC inhibitors, to date these agents have been disappointing in solid tumours clinically. The reasons for this are not completely clear, and may be due to the validity and clinical relevance of HDAC as a therapeutic target. However, other factors may also be important. For example, the level and duration of target inhibition may be suboptimal and the patient populations or tumour types studied may not have been appropriately selected. All of these factors are important to consider when developing drugs in this class, given that it is a crowded field with at least two already approved agents.

With these factors in mind, phase II trials utilising SB939 as monotherapy in translocation-associated sarcoma and prostate cancer patients are underway. The rationale for SB939 treatment in translocation-associated sarcomas is perhaps the best explained in the synovial sarcoma, where fusions of the *SS18* and *SSX (1, 2* and *4)* genes are characteristic. These gene fusions are associated with histone deacetylation and subsequent repression of the tumour suppressor gene *EGR1* (Soulez *et al*, 1999; van der Vlag and Otte, 1999; Yochum and Ayer, 2001; Furuyama *et al*, 2004; Terry *et al*, 2007). *In vivo* studies have shown that the use of HDAC inhibitors leads to the reversal of the above aberrances (i.e., acetylation of histones with restoration of *EGR1* expression) and subsequently results in cell death (Lubieniecka *et al*, 2008). Meanwhile, the *TMPRSS2-ERG* translocation, which has been found to occur with regular frequency in prostate carcinomas, appears to be associated with HDAC expression and is thus linked with sensitivity to HDAC inhibitors (Tomlins *et al*, 2005; Iljin *et al*, 2006; Bjorkman *et al*, 2008). Additionally, further studies with SB939, especially in combination with other cytotoxic and or targeted therapies, will help define the relevant treatment schedule and best potential anti-tumour activity in specific tumour types.

## Figures and Tables

**Table 1:** Patient characteristics

**Characteristics**	**No.**	**%^a^**
*No. of treated patients*	38	
Median age, years	62	
Range	20–88	
		
*Sex*		
Male	23	61
Female	15	39
		
*ECOG PS*		
0	7	18
1	26	68
2	5	13
		
*Primary tumour site*		
Colon	15	39
Rectal	3	8
Neuroendocrine	3	8
Gastric	2	5
Head and neck	2	5
Non small cell lung cancer	2	5
Small bowel/appendix	2	5
Cervix	1	3
Renal	1	3
Melanoma	1	3
Ovary	1	3
Prostate	1	3
Salivary gland	1	3
Thyroid	1	3
Unknown primary/others	2	5
		
*Measurable disease*		
Yes	36	95
No	2	5
		
*Prior therapy*		
Chemotherapy	33	87
Hormone therapy	1	3
Immunotherapy	1	3
Radiotherapy	17	45
Other therapy	16	42
		
*No. of prior chemotherapy regimens*		
0	5	13
1	1	3
2	10	26
3	14	37
4	5	13
5	2	5
7	1	3

Abbreviations: ECOG=Eastern Cooperative Oncology Group; PS=performance status.

a Percentage values rounded to the nearest zero decimal point, therefore not all values add up to a 100%.

**Table 2:** Dose level evaluated and DLT encountered

**Dose level**	**Dose**	**Schedule**	**Total no. of cycles**	**No. of patients with DLT/total no. of patients treated**	**DLT**
1	10	3 days every 2 weeks	6	0/3	
2	10	5 days every 2 weeks	16	1/6^a^	
3	20	5 days every 2 weeks	20	1/6	Grade 3 myositis
4	30	5 days every 2 weeks	19	0/4	
5	50	5 days every 2 weeks	6	0/4	
6	70	5 days every 2 weeks	5	0/3	
7	90	5 days every 2 weeks	3	1/2^b^	Grade 3 fatigue Grade 3 vomiting
					
6	70	5 days every 2 weeks	4	3/3	Grade 4 platelets Grade 3 fatigue
5.5	60	5 days every 2 weeks	17	1/7^c^	Grade 3 ALT^d^

Abbreviations: DLT=dose-limiting toxicity; ALT=alanine transaminase.

a Grade 3 bilirubin subsequently declared not drug related.

b Neither patient completed a full cycle.

c One patient had intolerable G2 nausea vomiting resulting in failure to receive further dose. Although this is not DLT *per se* according to protocol definition, we have classified this event as a DLT due to the inability to resume treatment.

d One patient did not complete cycle 1 because of ALT rise, however, it was not clear if this was entirely related to drug since patient had elevated transaminases with prior treatment.

**Table 3:** Related haematological and biochemical adverse events of all grades and those grade 3 or higher^a^

**Dose level**	**1**		**2**		**3**		**4**		**5**		**5.5**		**6**		**7**	
**No. of patients**	**3**		**6**		**6**		**4**		**4**		**7**		**6**		**2**	
**Dose**	**10 mg^b^**		**10 mg**		**20 mg**		**30 mg**		**50 mg**		**60 mg**		**70 mg**		**90 mg**	
**Grades**	**All grades**	**Grades 3/4**	**All grade**	**Grades 3/4**	**All grades**	**Grades 3/4**	**All grades**	**Grades 3/4**	**All grades**	**Grades 3/4**	**All grades**	**Grades 3/4**	**All grades**	**Grades 3/4**	**All grades**	**Grades 3/4**
*General*																
Fatigue	0	0	2	0	2	0	1	0	3	0	6	1	4	2	2^c^	1
																
*GI*																
Anorexia	0	0	0	0	2	0	0	0	1	0	4	0	3	0	1	0
Altered taste	0	0	0	0	1	0	0	0	0	0	1	0	0	0	1	0
Mucositis	1	0	1	0	0	0	0	0	0	0	0	0	1	0	0	0
Nausea	0	0	0	0	2	0	0	0	2	0	5	0	4	0	2	0
Vomiting	0	0	0	0	1	0	0	0	2	0	4	0	2	0	2^c^	1
Diarrhoea	0	0	1	0	1	0	1	0	0	0	4	1	0	0	0	0
Constipation	0	0	0	0	0	0	0	0	0	0	0	0	0	0	0	0
Abdominal pain	0	0	0	0	0	0	0	0	0	0	0	0	0	0	0	0
																
																
*Cardiac*																
QT prolongation	0	0	1	0	0	0	0	0	0	0	1	0	1	0	1	0
Ventricular bigemini	0	0	1^c^	0	0	0	0	0	0	0	0	0	0	0	1	0
Ventricular arrythmias (PVCs)	0	0	1^c^	0	0	0	0	0	0	0	0	0	0	0	1	0
Cardiac dysfunction	0	0	0	0	0	0	0	0	0	0	0	0	0	0	1	0
Supra ventricular arrythmia	0	0	0	0	0	0	0	0	0	0	0	0	0	0	1	0
Cardiac ischaemia	0	0	0	0	0	0	0	0	0	0	0	0	0	0	1	0
Cardiac arrythmia other	0	0	0	0	0	0	0	0	0	0	1	0	0	0	1	0
																
*Metabolic*																
Creatinine (raised)	0	0	1	0	1	0	2	0	0	0	2	0	2	0	2	0
Alkaline phophatase (raised)	1	0	5	1	4	1	2	0	2	0	6	0	3	0	1	0
ALT (raised)	0	0	1	0	2	0	2	0	0	0	4	2	1	0	2	0
AST (raised)	1	0	3	0	3	0	1	0	2	0	5	0	2	0	2	0
Bilirubin (raised)	0	0	1	0	1	0	1	0	0	0	2	0	0	0	1	0
CPK	NA	NA	0	0	1	1	1	0	0	0	0	0	1	0	1	0
																
*Hematologic*																
Anaemia	2	0	6	0	5	1	4	0	4	0	7	0	6	0	2	0
Leukopenia	1	0	2	0	1	0	2	0	1	0	5	1	3	1	1	0
Neutropenia	1	1	1	0	1	0	2	0	1	0	5	0	3	1	1	0
Lymphopenia	0	0	5	2	2	0	2	0	4	2	6	3	4	3	2	0
Thrombocytopenia	0	0	2	0	1	0	0	0	0	0	5	0	6	1	2	0
																
*Others*																
Myositis	0	0	0	0	1	1	0	0	0	0	0	0	0	0	0	0
Syncope	0	0	0	0	1	1	0	0	0	0	0	0	0	0	0	0

Abbreviations: ALT=alanine transaminase, AST=aspartate transaminase, CPK=creatine phosphokinase; PVCs=premature ventricular contraction.

a Graded according to CTCAE version 3.0.

b 3 days every 2 weeks.

c One or more SAE.

**Table 4:** Full data on AUC_0−∞_, *T*_1/2_, *C*_max_ and *T*_max_ for SB939 at different dose levels

			**Day 1**		**Day 3 or 5**	
**Dose level**	**Dose (mg)**	**Parameter**	**Mean**	**s.d.**	**Mean**	**s.d.**
1	10^a^	AUC_0–∞_ (ng h ml^-1^)	126.7	23.9	147.3	32.6
		*T*_1/2_ (h)	7.1	0.2	8.3	0.7
		*C*_max_ (ng ml^-1^)	14.6	6.0	18.3	6.4
		*T*_max_ (h)	2.5	0.9	1.3	0.3
						
2	10	AUC_0–∞_ (ng h ml^-1^)	137.2	26.6	187.7	32.7
		*T*_1/2_ (h)	8.6	1.5	8.1	2.5
		*C*_max_ (ng ml^-1^)	19.5	8.1	23.3	11.4
		*T*_max_ (h)	1.1	0.6	1.3	0.6
						
3	20	AUC_0–∞_ (ng h ml^-1^)	243.7	117.1	424.0	235.4
		*T*_1/2_ (h)	5.9	2.4	7.7	0.6
		*C*_max_ (ng ml^-1^)	45.9	28.5	51.3	31.9
		*T*_max_ (h)	1.5	0.9	1.6	0.8
						
4	30	AUC_0–∞_ (ng h ml^-1^)	383.8	131.8	544.5	206.3
		*T*_1/2_ (h)	8.6	1.7	8.8	2.1
		*C*_max_ (ng ml^-1^)	55.8	21.7	74.3	28.9
		*T*_max_ (h)	1.8	0.9	1.6	0.5
						
5	50	AUC_0–∞_ (ng h ml^-1^)	661.3	415.3	793.7	11.2
		*T*_1/2_ (h)	5.6	1.3	8.9	1.2
		*C*_max_ (ng ml^-1^)	124.1	93.5	101.3	5.6
		*T*_max_ (h)	1.6	1.0	1.7	0.3
						
5.5	60	AUC_0–∞_ (ng h ml^-1^)	1028.7	229.4	1625.2	405.5
		*T*_1/2_ (h)	6.7	0.4	6.9	1.6
		*C*_max_ (ng ml^-1^)	209.6	55.3	262.5	65.9
		*T*_max_ (h)	1.2	0.5	1.3	0.4
						
6	70	AUC_0–∞_ (ng h ml^-1^)	1053.8	664.9	1134.3	678.0
		*T*_1/2_ (h)	7.0	1.7	6.4	0.7
		*C*_max_ (ng ml^-1^)	178.2	122.2	159.7	118.8
		*T*_max_ (h)	1.6	0.4	1.5	0.5
						
7	90	AUC_0–∞_ (ng h ml^-1^)	1130.0	43.8	1229.0	—
		*T*_1/2_ (h)	6.4	0.5	6.1	—
		*C*_max_ (ng ml^-1^)	228.9	7.1	116.0	—
		*T*_max_ (h)	1.0	0.0	1.5	—

a 3 days every 2 weeks.
